# Lithium for prevention of mood episodes in bipolar disorders: systematic review and meta-analysis

**DOI:** 10.1186/s40345-014-0015-8

**Published:** 2014-12-20

**Authors:** Emanuel Severus, Matthew J Taylor, Cathrin Sauer, Andrea Pfennig, Philipp Ritter, Michael Bauer, John R Geddes

**Affiliations:** Department of Psychiatry and Psychotherapy, University Hospital Carl Gustav Carus, Technische Universität Dresden, Dresden, Germany; Department of Psychosis Studies, Institute of Psychiatry, Psychology & Neuroscience, King’s College London, London, UK; Department of Psychiatry, University of Oxford, Oxford, UK

**Keywords:** Lithium, Placebo, Anticonvulsants, Systematic review, Randomized controlled trial, Meta-analysis, Bipolar disorders, Long-term treatment

## Abstract

**Background:**

In a previous meta-analysis of randomized controlled trials comparing lithium with placebo as a long-term treatment in bipolar disorders, we observed a clear preventative effect for manic episodes; however, the effect was equivocal for depressive episodes. Since then, the evidence base has grown further. In this update, we furthermore present the data on efficacy of lithium in comparison to alternative drug treatments. In addition, we analyze the data comparing lithium with placebo and other treatments regarding drop-outs due to reasons other than a mood episode and completion of study (no mood episode and no drop-out to reasons other than a mood episode).

**Methods:**

Randomized controlled trials (RCTs) were sought comparing lithium with placebo and lithium with an alternative treatment in bipolar disorders where the stated intent of treatment was prevention of mood episodes. To this purpose, the Cochrane Central Register of Controlled Trials (CENTRAL) was searched. Reference lists of relevant papers and major textbooks of mood disorders were examined. Authors, other experts in the field, and pharmaceutical companies were contacted for knowledge of suitable trials, published or unpublished.

**Results:**

For the comparison of lithium with placebo, seven trials (1,580 participants) were included. Lithium was more effective than placebo in preventing overall mood episodes (random effects RR 0.66, 95% CI 0.53 to 0.82), manic episodes (random effects RR 0.52, 95% CI 0.38 to 0.71), and, dependent on the type of analyses applied, depressive episodes (random effects RR 0.78, 95% CI 0.59 to 1.03; fixed effect RR 0.73, 95% CI 0.60 to 0.88). Lithium was inferior to placebo in leading to drop-outs for reasons other than a mood episode (random effects RR 1.33, 95% CI 1.07 to 1.65) but superior to placebo on study completion (random effects RR 1.69, 95% CI 1.12 to 2.55).

For the comparison of lithium with anticonvulsants, seven trials were included (*n* = 1,305). In prevention of manic episodes, lithium showed superiority compared to anticonvulsants (random effects RR 0.66, 95% CI 0.44 to 1.00). However, there was no significant difference regarding prevention of overall mood episodes, depressive episodes, dropping-out to reasons other than a mood episode, or study completion.

**Conclusions:**

The evidence base for lithium in the long-term treatment of bipolar disorders has strengthened. With no other drug available having such ample and consistent evidence for its efficacy lithium remains the most valuable treatment option in this indication.

## Background

### Rationale

Bipolar disorders are common and disabling and have a tendency to recur. The defining features of this group of disorders are disturbances of mood with episodes of depression (lowered mood) and mania (elevated/irritable mood and increased energy). Most episodes of illness recover over time and with treatment, but there is a marked tendency for these disorders to be recurrent (Geddes and Miklowitz 2013). It has been estimated that at least 80% who have an episode of mania will have one or more recurrences (NIMH/NIH Consensus Development Conference statement 1985; 2002; Quitkin et al. 1976). In addition, subclinical symptoms may persist and become chronic.

Long-term treatment to prevent mood episodes (relapse during continuation treatment as well as recurrences during maintenance/prophylactic treatment) is therefore of vital importance in the management of bipolar disorders. That lithium can prevent mood episodes has been recognized since the 1960s (Abou-Saleh and Coppen 1986). Since then, lithium has become a mainstay of preventative treatment in bipolar disorders, as well as in unipolar depression (Cipriani et al. 2006). Furthermore, lithium has been recommended for the treatment of acute mania and for the augmentation of antidepressants in unipolar depression (Katona 1995; Bauer et al. 2014). Its effectiveness as an antidepressant when used alone has been disputed (Bauer et al. 2006; Bschor et al. 2013). This review focuses on the use of lithium to prevent mood episodes in bipolar disorders.

Lithium is widely used in clinical practice in the long-term treatment of affective disorders (Kessing et al. 2011; Kessing et al. 2012). Its use has been considered well established (Licht 2012; Crossley et al. 2006), although some have seriously questioned its efficacy (Blackwell and Shepherd 1968; Moncrieff 1997). Abrupt discontinuation/lowering of long-term lithium treatment in bipolar patients is known to precipitate episodes of affective illness (Goodwin 1994; Suppes et al. 1991), in particular if serum levels abruptly drop of more than 0.2 mmol/L (Severus et al. 2008). The early randomized controlled trials that established the use of lithium therapy have been criticized as several of them were of a discontinuation design. It has been suggested that the considerable beneficial effect of lithium found in these studies was due to such a discontinuation effect (Moncrieff 1997).

In the latest previous systematic review and meta-analysis which dates back to 2004 (Geddes et al. 2004), we found evidence from randomized controlled trials including 770 participants that lithium treatment reduces the risk of mood episodes in bipolar disorders. The preventive effect was clear for manic episodes, although it was equivocal for depressive episodes. Since then, the evidence base has substantially grown further, particularly through lithium’s use as active comparator in trials of alternative treatments. The time is therefore ripe for a reassessment of the evidence for lithium’s efficacy in relapse/recurrence prevention of bipolar disorders. In addition, the previous meta-analysis did not include data on discontinuation for reasons other than a mood episode though differences between lithium and placebo/active comparator in this issue may bias the data on efficacy and vice versa.

### Objectives

Here, we aim to determine the efficacy of lithium therapy in preventing episodes of mood disorders in persons with bipolar disorders and to assess whether it is effective in the prevention of both manic and depressive episodes. In addition, we present data on study withdrawals - ‘drop-outs’ - due to reasons other than a mood episode (e.g. side effects) and number of individuals completing the study for lithium compared to placebo/alternative treatment. In addition to the methodological importance of study withdrawal (Licht and Severus 2014), scrutiny of withdrawal is important to provide a realistic idea on the effectiveness of lithium in real-world clinical practice: good tolerability is vital for long-term adherence which, in turn, is a prerequisite for lithium to realize its potential to prevent mood episodes and instability in bipolar disorders (Malhi et al. 2012).

## Methods

### Eligibility criteria

Randomized controlled parallel-group studies published in English or German were considered. Males and females aged 16+ with a diagnosis of bipolar disorders, in partial or full remission, were included. Studies of participants with mixed diagnoses of mood disorders were included where those participants with bipolar disorders were separately randomized between treatments.

### Types of interventions

- Studies included were those comparing lithium with placebo and those comparing lithium with an alternative treatment (anticonvulsant or atypical antipsychotic) to prevent mood episodes where follow-up was for at least 3 months.
- For the analyses comparing lithium with either an anticonvulsant or antipsychotic, we only included those comparisons where there were at least two studies for each specific drug as a smaller number was considered insufficient for estimating the between-studies variance.
- To increase the likelihood of comparability between intervention groups, we excluded small studies (*n* < 50 per treatment arm) where key data regarding potentially outcome-relevant clinical variables between the intervention groups at baseline were missing (i.e. age, sex, severity of the illness (history and at baseline)) (Pfennig et al. 2012).
- Discontinuation studies (in which patients who had been in remission on lithium for at least 2 consecutive months were selected, then randomly assigned to continued lithium treatment or placebo/alternative treatment substitution) were excluded from the analyses.
- We excluded trials that were confounded by adjunctive treatments i.e. when lithium was combined with another treatment such as an antidepressant or anticonvulsant. However, in factorial trials of lithium, placebo, and another active comparator, we included both the lithium versus placebo and the lithium versus active comparator comparison because the factorial design allows an unconfounded comparison.

### Types of outcome measures

We used the definition of relapse/recurrence as defined by the authors of the original trials (Table 1). We extracted and analyzed data on the total number of recurrences/relapses as well as the number of manic/hypomanic episodes and the number of depressive episodes. We extracted and analyzed the number of participants dropping out of treatment during the study period for reasons other than a mood episode, as this number is possibly reflecting, at least in part, tolerability or acceptability issues. We also extracted and analyzed the number of individuals completing a study which is defined by the number of individuals randomized to either lithium or placebo/alternative treatment minus the number of individuals who developed a mood episode minus the number of individuals who dropped out of the study for reasons other than a mood episode.

**Table 1:** **Characteristics of studies included in a meta-analysis of trials assessing the effectiveness of lithium for prevention of mood episodes in bipolar disorders**

**Study**	**Year**	**Comparator**	**Design**	**Participants**	**Interventions (with levels)**	**Definition of relapse/recurrence**	**Quality (rating)**	**Previous lithium/comparator use**	**Lithium/comparator serum level achieved**
Prien 1973	1973	Placebo	Random assignment, 2-year follow-up	Patients with manic depressive disorder, manic type (*n* = 205), age 17 to 60 years; most recent episode: manic	Lithium (0.5 to 1.4 mEq/l); placebo	Emergent manic or depressive attack measured on Global Affective Scale requiring hospitalization (severe relapse) or supplementary medication (moderate relapse); combined moderate and severe relapse rates used	Allocation concealment unclear (B); participants and clinical raters blinded to treatment allocation; treating physician not blinded (A)	Following remission of the acute manic episode and prior to discharge (time of randomization) patients were stabilized on lithium (0.5 to 1.4 mEq/l)	Median serum lithium level 0.7 mEq/l
Kane 1982	1982	Placebo	Random assignment, up to 2-year follow-up	Patients with bipolar II disorder (Research Diagnostic Criteria) (*n* = 22), age 18 to 65 years; patients had been euthymic for 6 months prior to entry into the study	Lithium (0.8 to 1.2 mEq/l); imipramine (100 to 150 mg per day); lithium plus imipramine; placebo	Emergent mood episode meeting Research Diagnostic Criteria for major depressive disorder for 1 week, minor depressive disorder for 4 weeks, manic episode for any duration, or hypomanic episode for 1 week	Allocation concealment unclear (B); patients and physicians blinded to treatment allocation (A)	Patients had been on open uncontrolled continuation treatment for 6 months (except for the last 6 weeks (open treatment with imipramine)) before they were randomly assigned to treatment condition	Not available
Greil 1997	1997	Carbamazepine	Random assignment, 2.5 years observation period, primary aim was to assess efficacy of carbamazepine	Patients with current episode of bipolar affective disorder (ICD-9: 296.2, 296.3, 296.4) (*n* = 144), no preventive treatment immediately before current episode, age 18 to 65 years	Lithium (0.6 to 0.8 mmol/l); carbamazepine (4 to 12 μg/ml)	Recurrence, i.e. rating of psychopathology of 5 (=recurrence) or 6 (=extremely severe recurrence) corresponding to the recurrence of an affective episode in line with the Research Diagnostic Criteria	Non-blind design, randomization procedure by Efron (1971) (A); Allocation concealment adequate (A): central allocation through coordinating study centre, treatment group allocation by phone at the moment of randomization	Stabilization phase: psychotropic medication according to the free decision of the treating physician was gradually reduced and, if possible, discontinued before randomization; 84% never had received prophylactic treatment before	0.63 ± 0.12 mmol/l
Bowden 2000	2000	Placebo, valproate	Random assignment, 1-year follow-up, primary aim was to assess efficacy of divalproex	Patients with bipolar I disorder (DSM-III-R) with index manic episode according to Structured Clinical Interview for DSM-III-R (n=372), those with high suicide risk excluded, age 18-75 years	Lithium (0.8 to 1.2 mEq/l); divalproex (71 to 125 ug/ml); placebo	Emergent manic episode (Mania Rating Scale score of 16 or more or requiring hospitalization) or depressive episode (requiring antidepressant use or premature study withdrawal)	Allocation concealment unclear (B); patients, clinicians, and outcome assessors blinded to treatment allocation (A)	Before randomization, 117/372 were treated with open-label divalproex, 124 with lithium, 50 with both drugs, 81 with neither drug; lithium as well as divalproex were gradually reduced and withdrawn during the first 2 weeks of maintenance treatment	Mean (SD) serum lithium concentration by day 30: 1.0 ± 0.48 mEq/l; mean (SD) valproate concentration by day 30: 84.8 ± 29.9 ug/ml
Bowden 2003	2003	Placebo, lamotrigine	Random assignment, 1-year follow-up, primary aim was to assess efficacy of lamotrigine	Patients with bipolar I disorder recently recovered from a manic or hypomanic episode (DSM-IV) (*n* = 175), age ≥18 years	Lithium (0.8 to 1.1 mEq/l); placebo; lamotrigine (100 to 400 mg per day)	Intervention (additional medication or ECT) required for any mood episode; secondary outcomes subdivided by type of mood episode (manic/hypomanic/mixed or depressive)	Allocation concealment unclear (B); patients, clinicians, and outcome assessors blinded to treatment allocation (A)	Majority of participants had a prior history of lithium use (31/46: 70% in lithium group; 42/69: 67% in placebo group; 38/58: 72% in lamotrigine group). 18% of participants during the initial part of the 8- to 16-week open-label phase received lithium, the dosage of which was tapered over at least 3 weeks and discontinued a minimum of 1 week before they entered the double-blind phase of the study. All participants received open-label lamotrigine during the open-label phase (target dosage 200 mg/d; minimum 100 mg/d). Concomitant psychotropic medications were permitted during the open-label phase as needed to treat an ongoing manic or hypomanic episode but were discontinued a minimum of 1 to 2 weeks before entry into the double-blind phase.	Not available
Calabrese 2003	2003	Placebo, lamotrigine	Random assignment, 1-year follow-up, primary aim was to assess efficacy of lamotrigine	Patients with bipolar I disorder recently recovered from a major depressive episode according to DSM-IV (*n* = 463), age ≥18 years	Lithium (0.8 to 1.1 mEq/l); placebo; lamotrigine (50 to 400 mg per day)	Intervention (additional medication or ECT) required for any mood episode; secondary outcomes subdivided by type of mood episode (manic/hypomanic/mixed or depressive)	Allocation concealment unclear (B); patients, clinicians, and outcome assessors blinded to treatment allocation (A)	Majority of participants had a prior history of lithium use (57% to 62% of patients had received prior lithium treatment at some point, with 67% to 72% of these patients having achieved good clinical response and 80% to 85% having tolerated such prior treatment). 20% of participants in open label run in received lithium, dosage tapered over at least 3 weeks and discontinued a minimum of 1 week prior to entering the double-blind phase; any psychotropic medication permitted during 8- to 16- week open-label phase; all patients received lamotrigine (target dosage 200 mg/d; minimum 100 mg/d) as adjunctive therapy or monotherapy; all psychotropic medication other than lamotrigine were discontinued at least 7 days prior to randomization	Steady-state mean ± SD serum levels of 0.8 ± 0.3 mEq/l
Hartong 2003	2003	Carbamazepine	Random assignment, 2-year study	Patients with bipolar disorder (DSM-III-R) with at least two episodes during the last 3 years, recovered from last episode (*n* = 94), age ≥18 years	Lithium (0.6 to 1.0 mmol/l); carbamazepine (6 to 10 mg/l))	Recurrence of an episode of (hypo)mania or major depression according to DSM-III-R criteria	Allocation concealment adequate: pharmacy-controlled block randomization (A); double dummy design, double blind (A)	Total lithium/carbamazepine treatment during lifetime ≤6 months; at randomization, no patient received antidepressants, antipsychotics, or benzodiazepines.	Lithium level mean (SD): 0.75 (0.18) mmol/l; carbamazepine level mean (SD): 6.8 (1.2) mg/l
Geddes 2010	2010	Valproate	Random assignment, 24-month follow-up, primary aim was to assess efficacy of lithium-valproate combination therapy	Patients with bipolar I disorder on the basis of a previous episode of mania meeting DSM-IV criteria (*n* = 330), age ≥16 years; most recent episodes 52% mania, 34% depression, 12% mixed, 3% cycling	Lithium (0.4 to 1.0 mmol/l); valproate (750 to 1,250 mg)	Initiation of new intervention for an emergent mood episode, including drug treatment or admission to hospital	Randomization computerized, minimization; allocation concealment adequate: central allocation via telephone (A); investigators and participants informed of treatment allocation, trial management team masked to treatment assignment (A)	Before randomization active run-in of 4 to 8 weeks: all patients received lithium and valproate (lithium serum level 0.4 to 1.0 mmol/l; valproate dose at least 750 mg or valproic acid serum concentration at least 50 μg/ml)	Not available
Licht 2010	2010	Lamotrigine	Random assignment, up to 5.8-year follow-up, primary aim was to assess efficacy of lamotrigine	Patients with bipolar I disorder according to DSM-IV with at least two episodes within the last 5 years (*n* = 155) recruited during or in the aftermath of an index episode, age ≥18 years; index episode either depression (51%), mania (41%), or mixed mania (8%) according to the Cincinnati criteria, onset within the last year prior to randomization	Lithium (0.5 to 1.0 mmol/l); lamotrigine (up-titrated to 400 mg/day)	Psychotropic treatment (in addition to study drug and benzodiazepines) and/or hospitalization still required at month 6 after randomization; psychotropic treatment (in addition to study drug and benzodiazepines) during at least 1 week and/or hospitalization during at least 1 week still required after month 6 (after randomization)	Allocation concealment adequate: central allocation; computer-generated randomization plan, block randomization (A)	Prior lithium prophylaxis: 13 (17%) in lamotrigine group, 15 (19%) in lithium group; patients receiving lithium until randomization and assigned to lamotrigine group: lithium was tapered off over 1 to 3 months; patients receiving lamotrigine until randomization and assigned to lithium group: discontinuation of lamotrigine at randomization. Additional antipsychotic or antidepressant drugs were allowed in the first 6 months after randomization, investigators were encouraged to achieve monotherapy at month 6. Benzodiazepines allowed throughout the study.	Serum lithium level: mean 0.69 mmol/l (SD = 0.20); lamotrigine dose: mean 379 mg (SD = 66) (serum level 22.5 (12.7) μmol/l)
Amsterdam 2010	2010	Placebo, fluoxetine	Random assignment, up to 1-year follow-up	Patients with bipolar II disorder (*n* = 81) recently recovered from depressive episode with fluoxetine treatment, age 19 to 67 years	Lithium (0.5 to 1.5 mEq/l); fluoxetine (10 to 40 mg per day); placebo	Depressive relapse defined as HAMD score of 14 or more and meeting diagnostic criteria for major depressive episode. Hypomanic episode defined by DSM-IV criteria	Allocation concealment unclear (B); patients, clinicians, and outcome assessors blinded to treatment allocation (A)	Initial fluoxetine monotherapy was administered on the basis of response and tolerability. Patients who had a final HAM-D score ≤ 8 by week 12 of treatment were randomly assigned to different treatment arms. Patients assigned to fluoxetine group who previously took >40 mg/day of fluoxetine: dosage reduced to 40 mg/day; previously ≤ 40 mg/day: dosage maintained; patients assigned to lithium group: fluoxetine therapy discontinued. Lithium therapy initiated at 600 mg/day for 1 week, increased to 900 mg/day in week 2, continued until serum level of 0.5 to 1.5 mEq/l achieved by week 4.	Mean average serum lithium level was 0.69 mmol/liter (SD = 0.27), mean average maximum fluoxetine dose 34.3 mg/day (SD = 7.9)
Weisler et al. 2011	2011	Placebo, quetiapine	Random assignment, up to 2-year follow-up	Patients with bipolar I disorder (DSM-IV) recently recovered from a manic (53.6%), depressive (28%) or mixed episode (18.4%) (n = 1,172), age ≥18 years	Lithium (0.6 to 1.2 mEq/l); quetiapine (300 to 800 mg per day); placebo	Emergent mood event requiring medication or hospitalization, YMRS or MADRS 20 or more on two consecutive assessments, discontinuation attributed to mood event by investigator	Allocation by centralized randomization and drug allocation system (A); patients, clinicians, and outcome assessors blinded to treatment allocation (A)	All patients received open-label quetiapine (300 - 800 mg/d) for 4-24 weeks. Patients achieving stabilization on quetiapine were randomized to different treatment arms. Replacement of quetiapine tablets used during prerandomization phase started on day 1 and was completed by 2 weeks. Known intolerance or lack of response to lithium was an exclusion criterion	Mean (SD) median serum concentration was 0.63 (0.45) mEq/l; mean (SD) median quetiapine dose 546 (173) mg

### Information sources and search

1. Electronic databases
  The Cochrane Central Register of Controlled Trials (CENTRAL) were searched to April 2013 using the following search terms: (LITHIUM OR CAMCOLIT OR CARBOLITH OR DUROLITH OR ESKALITH OR LICARBIUM OR LISKONUM OR LITAREX OR LITHANE OR LITHOCARB OR LITHIZINE OR LITHONATE OR LITHOTABS OR MANIALITH OR PHASAL OR PRIADEL OR QUILONORM OR QUILONUM OR LI-LIQUID) and (bipolar OR mania OR manic). CENTRAL includes relevant records retrieved from MEDLINE, Embase, PsycINFO, Cochrane Review Group registers incorporating additional databases, and hand-searching activities.
2. Reference checking
  The reference lists of all identified randomized controlled trials, other relevant papers, and major textbooks on mood disorders were checked.
3. Hand searching
  The journals Lithium (1990 to 1994) and Lithium Therapy Monographs (1987 to 1991) were hand-searched.
4. Personal communication
  The authors of randomized controlled trials included in the review and other recognized experts in the field were contacted and asked if they had knowledge of any other studies, published or unpublished, relevant to the review. Pharmaceutical companies marketing lithium products were requested to provide relevant published and unpublished data. Following the publication of the first version of this review, we kept in contact with identified active trial lists and companies to identify any emerging trials. For one study (Licht et al. 2010), it was possible to add the split data for manic and depressive episodes (after 2 years of follow-up) to the review/meta-analysis after consulting the author.

### Study selection and data collection process

Studies generated by the search strategies were checked to ensure they met the previously defined inclusion criteria. Two reviewers independently extracted data concerning participant characteristics, intervention details (including participants’ lithium exposure immediately preceding the trial), and outcome measures from the included studies. Any disagreements were resolved by consensus.

### Risk of bias in individual studies

Quality assessment: the methodological quality of the included studies was assessed according to the Cochrane criteria for quality assessment (Higgins et al. 2003). On this basis, studies were given a rating of A (adequate randomization and concealment), B (unclear), and C (inadequate). Other aspects of methodological quality that have been shown to be related to validity were assessed by two reviewers independently. In cases where inadequate details of randomization and other methodologies were provided in published papers, the authors were contacted to obtain further information. Quality ratings were revised in several cases on the basis of information received from authors (Table 1).

### Summary measures and synthesis of results

Data analysis: data were analyzed using RevMan 5.1 software (The Cochrane Collaboration 2014) and R (R Development Core Team. R 2005). Heterogeneity between studies was assessed using the I^2^ statistic (Higgins et al. 2003).

For binary efficacy outcomes, random effects (DerSimonian and Laird 1986) and fixed effect (Greenland and Robins 1985; Mantel and Haenszel 1959) risk ratios with 95% confidence intervals were calculated. Fixed effect analysis assumes the included studies to be functionally identical and the underlying treatment effect size to be the same in all studies. Random effects analysis assumes a range of treatment effects and incorporates inter-study variation into the pooled estimate. Therefore, we primarily used the random effects model for our analyses. Nevertheless, in order to allow an estimation of the sensitivity of the results to the choice of method, we always present both sets of results. In addition, it is also common to report fixed effects if the statistical test for heterogeneity indicates relative homogeneity.

Where possible, we intended to use intention-to-treat (ITT) data for the primary efficacy analyses. Where ITT data were not available, we used endpoint data for trial completers. Data from trials including both unipolar and bipolar participants were only included in the respective analysis if the two diagnostic groups had been randomized separately.

## Results

### Study selection

**Figure 1:**
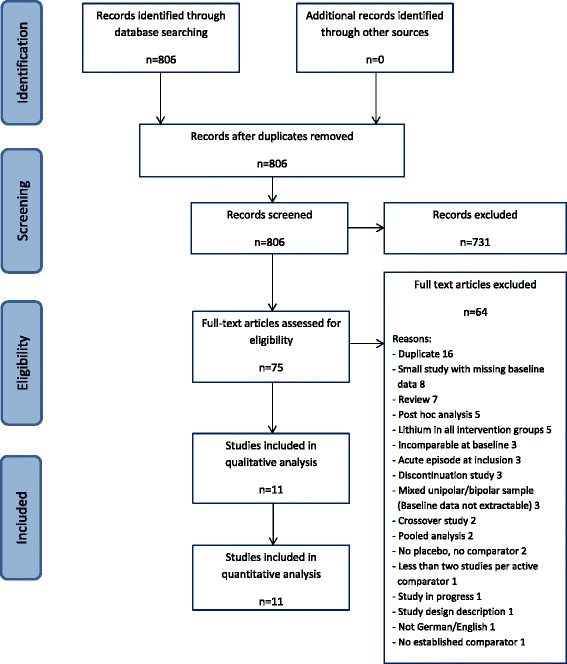
**PRISMA flowchart of the inclusion procedure.**

Using our search strategy, we identified 806 studies, which were subsequently screened. As a result, 731 records were excluded, while the remaining 75 records were assessed for eligibility (full-text articles). For a variety of reasons, as detailed in Figure 1, 11 studies could be included for qualitative and quantitative analysis (Moher et al. 2009).

### Study characteristics

#### Lithium versus placebo

Seven trials were included in this part of the updated review. Six trials included only bipolar participants (Prien et al. 1973; Bowden et al. 2000; Calabrese et al. 2003; Bowden et al. 2003; Amsterdam and Shults 2010; Weisler et al. 2011). One trial reported on groups with bipolar disorders and unipolar disorders that were randomized separately (Kane et al. 1982). Two trials were similar in design except that one recruited patients who had recently recovered from a depressive episode (Calabrese et al. 2003) and the other recruited patients who had recently recovered from a manic or hypomanic episode (Bowden et al. 2003).

Several trials included a third active treatment arm. In one study (Bowden et al. 2000), the third group received divalproex, a form of valproic acid; in two studies (Calabrese et al. 2003; Bowden et al. 2003), a third arm were allocated to lamotrigine. These three studies were also included in the lithium versus anticonvulsant analysis. One study included a fluoxetine arm (Amsterdam and Shults 2010) and the final study included a quetiapine arm (Weisler et al. 2011). One trial had a factorial design in which patients were allocated to lithium, placebo, imipramine, or lithium + imipramine (Kane et al. 1982). As stated in the ‘Methods’ section, we had decided to exclude treatment groups that were confounded by adjunctive antidepressant treatment, therefore we excluded the lithium + imipramine and the placebo + imipramine groups from the analyses.

#### Lithium versus anticonvulsant

Seven trials were included in this analysis, including the three studies from the lithium versus placebo analysis with an anticonvulsant as a third treatment arm (Bowden et al. 2000; Calabrese et al. 2003; Bowden et al. 2003).

Two studies compared lithium to carbamazepine (Greil et al. 1997; Hartong et al. 2003), three to lamotrigine (Licht et al. 2010; Calabrese et al. 2003; Bowden et al. 2003), and two studies to valproate (Bowden et al. 2000; Geddes et al. 2010). All trials included bipolar patients exclusively.

Two studies (Weisler et al. 2011; Tohen et al. 2005) were found that compared lithium to an atypical antipsychotic (quetiapine and olanzapine, respectively). According to the study inclusion criteria, we decided not to include an extra review combining these two studies at this time.

### Prior treatment stabilization

In one of the trials, all the participants were stabilized on lithium treatment for unstated lengths of time prior to randomization (Prien et al. 1973). In the study by (Bowden et al. 2000), 34% of the group allocated to lithium and 35% of the group allocated to placebo received lithium as an open treatment prior to randomization. In this study, lithium was discontinued gradually over two weeks in those participants allocated to placebo.

In two studies (Calabrese et al. 2003; Bowden et al. 2003) for those patients continuing ongoing lithium during the open-label phase, the dosage was tapered over at least 3 weeks and discontinued a minimum of 1 week prior to entering the double-blind phase of the study.

The studies followed participants from randomization either until they experienced a mood episode or for maximum periods of between 1 and 2 years.

The range of lithium levels employed was known for the trials and the ranges targeted were all between 0.4 and 1.5. mEq/l. In one trial, participants randomized to lithium but with inadequate serum lithium monitoring (54 of 418) were excluded from analysis (Weisler et al. 2011).

Five trials (Bowden et al. 2000; Calabrese et al. 2003; Bowden et al. 2003; Weisler et al. 2011; Geddes et al. 2010) stated that they included participants who have bipolar I disorder. Prien (Prien et al. 1973) required patients to have had a manic episode. Two trials included participants with bipolar II disorder (Amsterdam and Shults 2010; Kane et al. 1982).

### Methodological quality of included studies

The more recent trials (Bowden et al. 2000; Calabrese et al. 2003; Bowden et al. 2003; Weisler et al. 2011) overcome many of the methodological weaknesses of the older trials of lithium that have been described in the past such as lack of intention-to-treat analysis, masking of treatment allocation, size, diagnosis, and discontinuation artifacts (Burgess et al. 2001). However, the descriptions of the method of treatment allocation procedures and allocation concealment often remained inadequate.

## Synthesis of results

### Lithium versus placebo

#### Mood episode prevention

**Figure 2:**
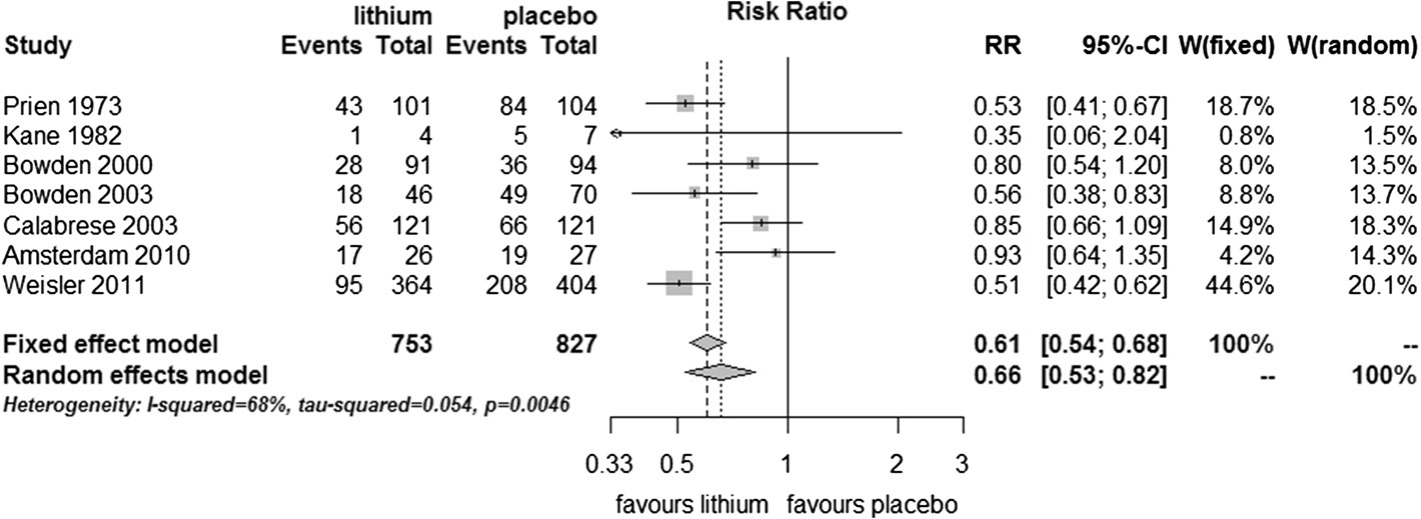
**Prevention of any episode in bipolar disorders patients in RCTs comparing lithium with placebo.**

Data were available from all seven trials with a total of 1,580 participants. Lithium was found to be more effective than placebo in preventing new episodes in bipolar disorders (fixed effect RR 0.61, 95% CI 0.54 to 0.68, I^2^ 68%; test for overall effect *p* < 0.001; random effects RR 0.66, 95% CI 0.53 to 0.82, test for overall effect *p* < 0.001). Although moderate to high statistical heterogeneity was seen, the direction of effect was the same in all trials: no trial was found that lithium is inferior to placebo (Figure 2).

**Figure 3:**
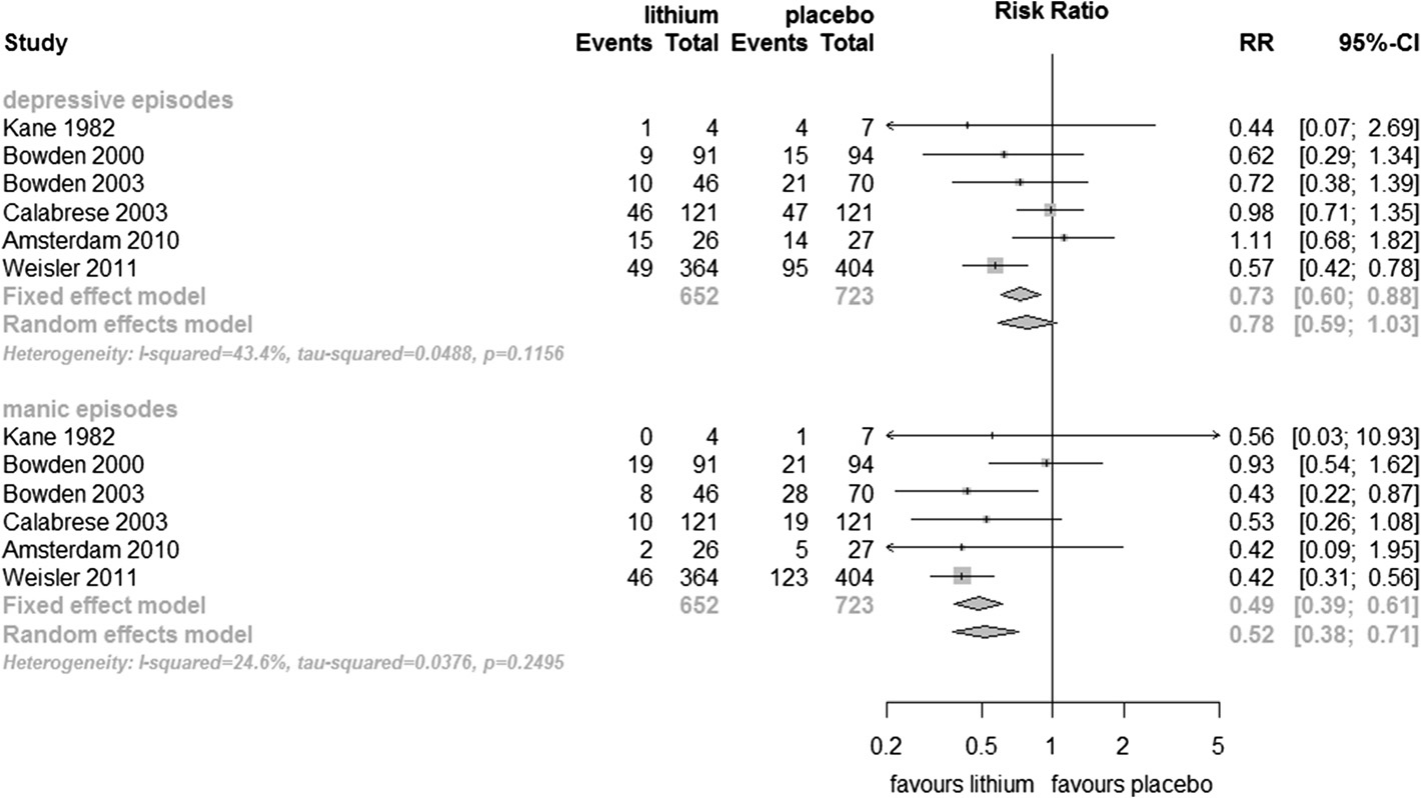
**Prevention of depressive/manic episodes in bipolar disorders patients in RCTs comparing lithium with placebo.**

Data on relapses/recurrences into mania and depressive episodes were available separately from six trials (1,375 participants). Lithium prevented manic/hypomanic episodes (fixed effect RR 0.49, 95% Cl 0.39 to 0.61, I^2^ 25%; test for overall effect *p* < 0.001; random effects RR 0.52, 95% CI 0.38 to 0.71, test for overall effect *p* < 0.001). Lithium prevented depressive episodes in a fixed effect analysis (fixed effect RR 0.73, 95% CI 0.60 to 0.88, I^2^ 49%; test for overall effect *p* < 0.001). Random effects analysis just failed to reach conventional statistical significance (random effects RR 0.78, 95% CI 0.59 to 1.03, test for overall effect *p* = 0.08). Heterogeneity was not statistically significant in these analyses (Figure 3).

#### Discontinuation of treatment due to reasons other than a mood episode

**Figure 4:**
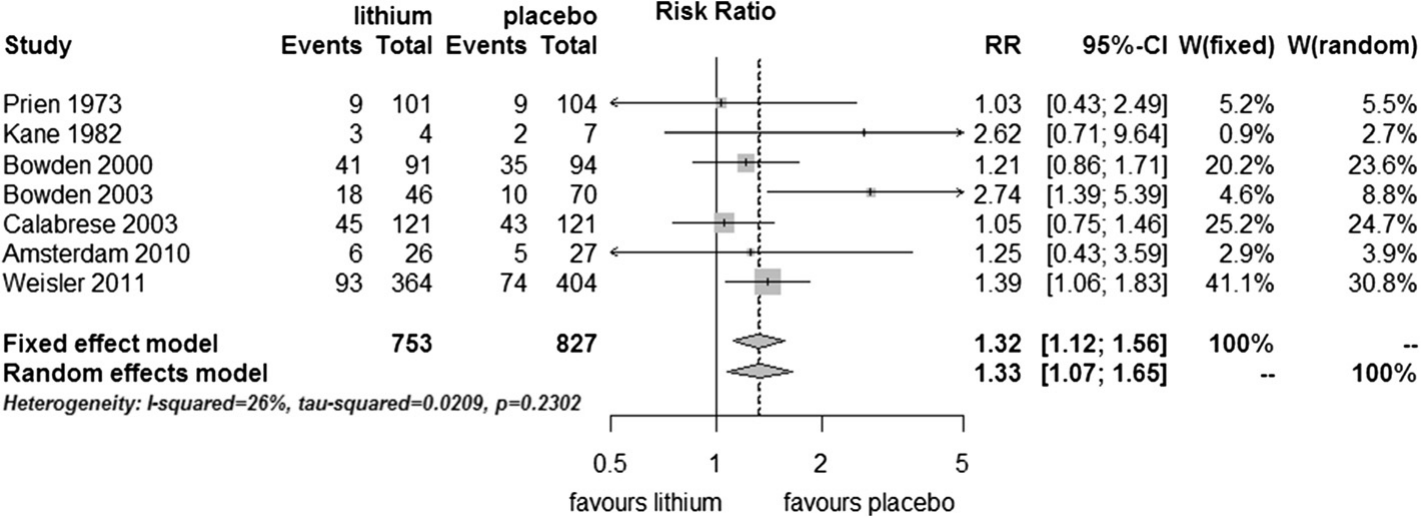
**Discontinuation of study due to reasons other than a mood episode in bipolar disorders patients in RCTs comparing lithium with placebo.**

There were significantly more dropouts - discontinuation for reasons other than mood episode - in those treated with lithium compared to placebo (fixed effect RR 1.32, 95% CI 1.12 to 1.56; I^2^ 23%, test for overall effect *p* = 0.001; random effects RR 1.33, 95% CI 1.07 to 1.65, test for overall effect *p* = 0.01) (Figure 4).

#### Study completion

**Figure 5:**
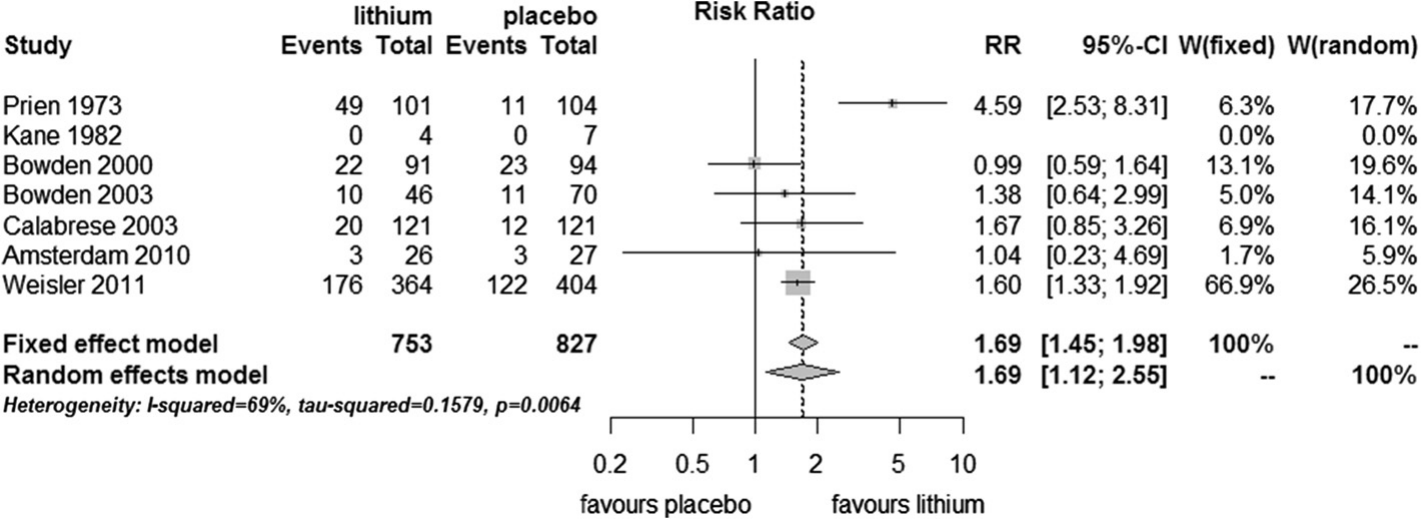
**Study completion in bipolar disorders patients in RCTs comparing lithium with placebo.**

Significantly, more patients completed the trials without an episode or drop out in the group receiving lithium compared with placebo (fixed effect RR 1.69, 95% CI 1.45 to 1.98, I^2^ 69%, test for overall effect *p* < 0.001; random effects RR 1.69, 95% CI 1.12 to 2.55, test for overall effect *p* = 0.01) (Figure 5).

### Lithium versus anticonvulsants

#### Mood episode prevention

**Figure 6:**
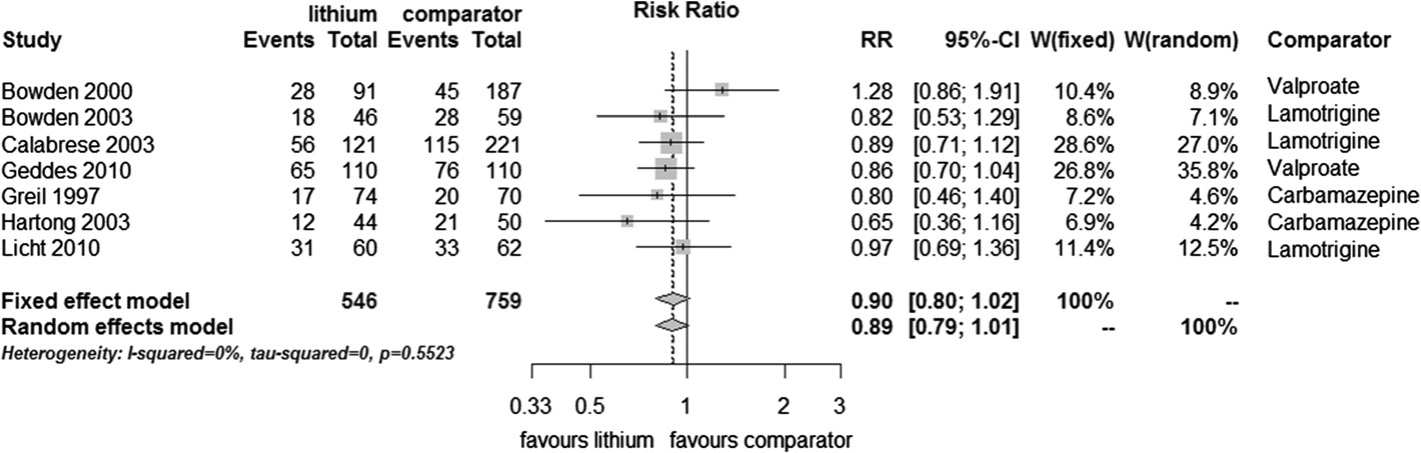
**Prevention of any episode in bipolar disorders patients in RCTs comparing lithium with anticonvulsants.**

There was available data from seven trials (1,305 participants). Fewer participants on lithium relapsed than on anticonvulsant, although the difference did not meet conventional levels of statistical significance in either fixed effect (RR 0.90, 95% CI 0.80 to 1.02, I^2^ 0%, test for overall effect *p* = 0.10) or random effects analysis (random effects RR 0.89, 95% CI 0.79 to 1.01, test for overall effect *p* = 0.07). Heterogeneity was not significant (Figure 6).

**Figure 7:**
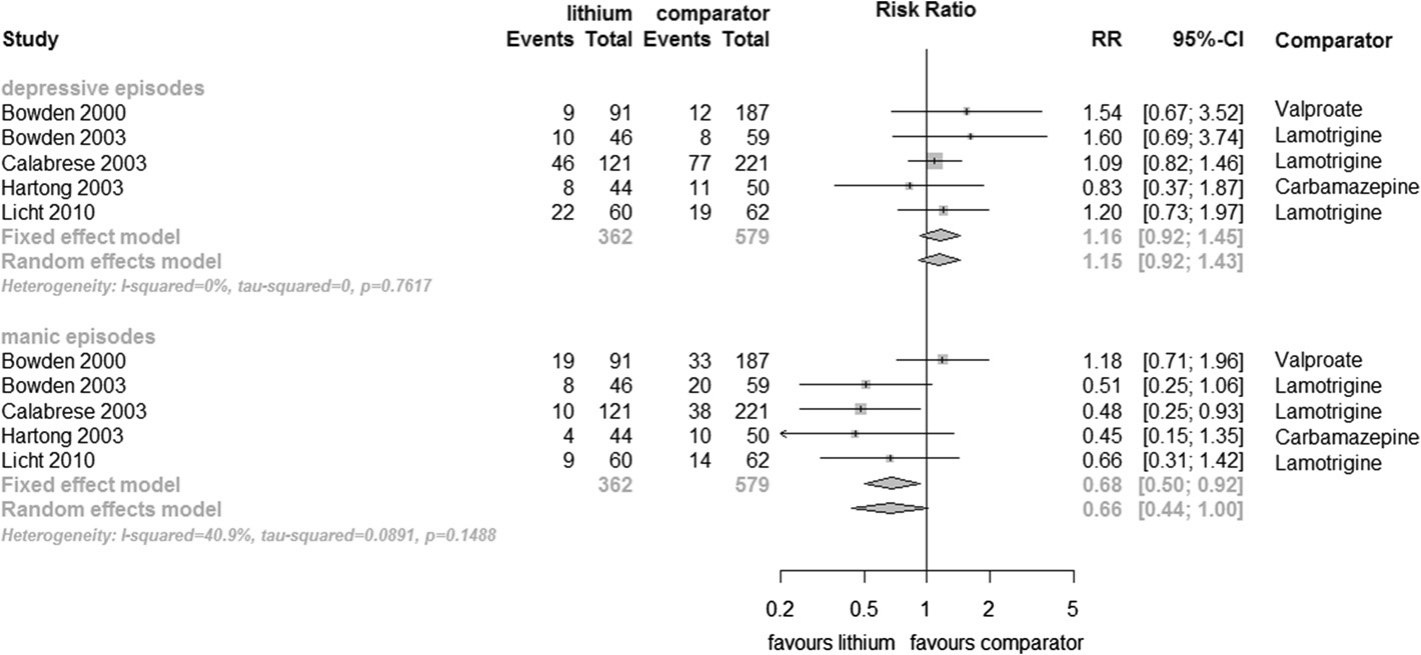
**Prevention of depressive/manic episodes in bipolar disorders patients in RCTs comparing lithium with anticonvulsants.**

Data for manic and depressive episodes separately were available from five of the seven trials, (*n* = 941). Lithium showed significant superiority over anticonvulsants in the prevention of manic episodes (fixed effect RR 0.68, 95% CI 0.50 to 0.92, I^2^ 41%, test for overall effect *p* = 0.01, random effects RR 0.66, 95% CI 0.44 to 1.00, test for overall effect *p* = 0.05). There was no significant difference between lithium and anticonvulsant in the prevention of depressive episode (fixed effect RR 1.16, 95% CI 0.92 to 1.45, I^2^ 0%, test for overall effect *p* = 0.20, random effects RR 1.15, 95% CI 0.92 to 1.43, test for overall effect *p* = 0.23) (Figure 7).

#### Discontinuation of treatment due to reasons other than a mood episode

**Figure 8:**
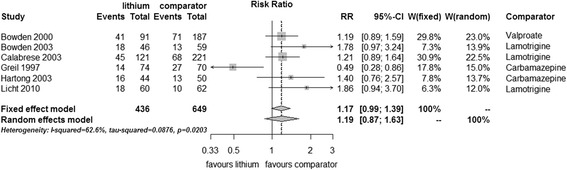
**Discontinuation of study due to reasons other than a mood episode in bipolar disorders patients in RCTs comparing lithium with anticonvulsants.**

Data for discontinuation for reasons other than a mood episode were available from six studies (*n* = 1,085). There was significant heterogeneity (I^2^ 62.6%, *p* = 0.02), and no significant difference between lithium and anticonvulsants was found (fixed effect RR 1.17, 95% CI 0.99 to 1.39, I^2^ 63%, test for overall effect *p* = 0.07, random effects RR 1.19, 95% CI 0.87 to 1.63, test for overall effect *p* = 0.27) (Figure 8).

#### Study completion

**Figure 9:**
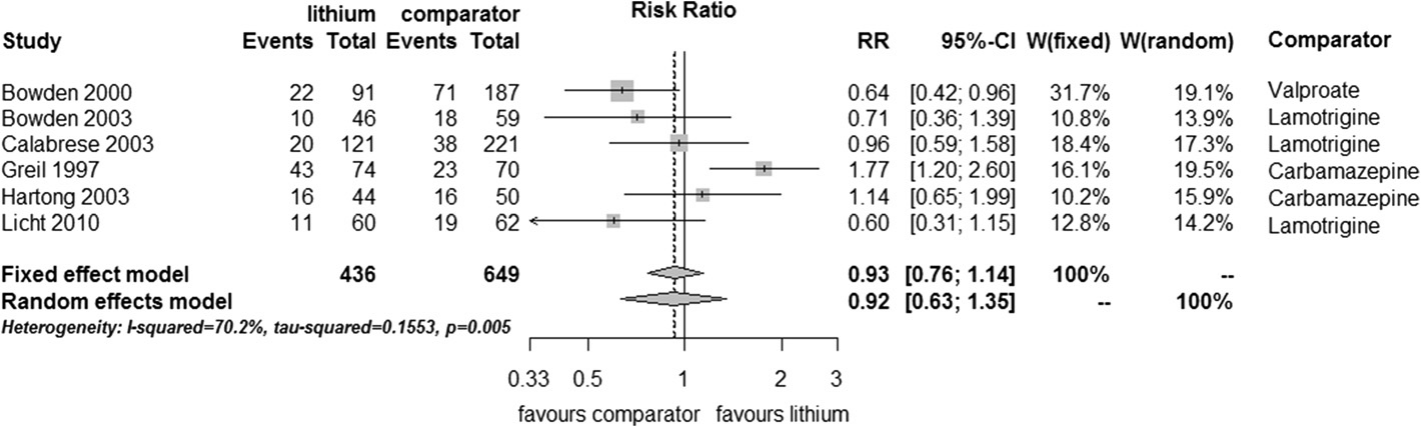
**Study completion in bipolar disorders patients in RCTs comparing lithium with anticonvulsants.**

There was no significant difference in the number of completers (fixed effect RR 0.93, 95% CI 0.76 to 1.14, I^2^ 70%, test for overall effect *p* = 0.50, random effects RR 0.92, 95% CI 0.63 to 1.35, test for overall effect *p* = 0.67) (Figure 9).

## Discussion

### Summary of evidence

This systematic review shows that treatment with lithium decreases the probability of mood episodes compared to placebo for up to 2 years in patients with bipolar disorders. The treatment effect is present for prevention of both manic relapse/recurrence and depressive relapse/recurrence, with the statistical significance of the latter finding dependent on the type of analysis performed. The total number of participants has increased substantially over the past decade, and the more recent trials overcome many of the shortcomings of earlier trials. In the analysis of overall mood episode rates, there was evidence of statistically significant heterogeneity between trials, but no single trial found lithium to be less effective than placebo.

In all of the included trials except Prien et al. (1973) and Geddes et al. (2010), data from patients who developed a mood episode were censored from the analysis of time to discontinuation for reasons other than a mood episode (mutually exclusive endpoints). Similarly, data from patients who developed a depressive episode were censored from the analyses of time to a manic episode - and vice versa. In contrast to Kaplan–Meier survival analyses, in meta-analyses, with categorical data used for analyses, censoring due to a mood episode may substantially affect the risk of discontinuation due to reasons other than a mood episode - and vice versa - if the drug affects the risk of a mood episode compared to the risk of dropping out due to reasons other than a mood episode differently than placebo. For lithium, this seems to be the case. In our meta-analysis, significantly fewer patients on lithium compared to placebo developed a mood episode while at the same time, significantly more patients on lithium compared to placebo dropped out of the study due to reasons other than a mood episode. Assuming that lithium’s prophylactic efficacy against mood episodes were the primary event, more patients would be at risk to potentially drop out for reasons other than a mood episode (for example, due to side effects) - and consequently, this meta-analysis may be biased to the disadvantage of lithium when it comes to dropping out of the study for reasons other than a mood episode. Therefore, studying the number of individuals who completed the study without dropping out for whatever reasons (mood episodes + reasons other than a mood episode) is not only clinically highly relevant - as lithium can only work if patients adhere to it - but also essential to get an unbiased picture on how lithium compares to placebo in the long-term treatment of bipolar disorders. In the present meta-analysis, lithium proved superior to placebo in the number of individuals who completed the study, thus confirming the results from the analyses of mood episodes. However, there was significant quantitative heterogeneity.

Regarding the polarity of new mood episodes, the relative risk reduction of lithium appears more substantial against manic episodes; however, the same limitations which apply to data censoring due to mood episodes and reasons other than a mood episode also apply in this case (Licht and Severus 2014). In addition, the majority of patients in this meta-analysis were recruited with an index manic episode which increases the likelihood of a manic versus depressive recurrence/relapse - and therefore by itself increases the probability of establishing prophylactic antimanic versus antidepressant efficacy of a given drug, all other relevant factors being equally distributed (Calabrese et al. 2004). Therefore, lithium may even perform better regarding depressive episodes than this meta-analysis suggests. Furthermore, given the higher absolute risk of depressive episodes (Judd et al. 2002), lithium probably does protect against depression to a clinically worthwhile degree, similar to recurrent unipolar depression (Cipriani et al. 2006). When manic and depressive episodes were considered separately, there was no statistically significant heterogeneity between the trials.

### Limitations

#### Limitations at study and outcome level

While this meta-analysis deals with the efficacy of lithium to prevent mood episode in bipolar disorders, it has to be said that the majority of the trials upon which this meta-analysis is based primarily deals with patients suffering from bipolar I disorder, with only two trials exclusively enrolling patients with bipolar II disorder (Amsterdam and Shults 2010; Kane et al. 1982) - and two further including a small percentage of patients with bipolar II disorder (Greil et al. 1997; Hartong et al. 2003). Therefore, our results primarily apply to patients with bipolar I disorder.

To get an unbiased picture of lithium’s efficacy in the long-term treatment of bipolar disorders, we decided to exclude lithium discontinuation studies in which patients with bipolar disorders who had been in remission on lithium for at least 2 consecutive months before being randomized to either continued lithium treatment or placebo/active comparator were selected (Coxhead et al. 1992; Melia 1968; Wolf et al. 1997). As a rule, patients who have been in remission for at least 2 consecutive months are generally believed to have recovered from the index episode and enter prophylactic treatment (Grunze et al. 2013; Tohen et al. 2009). Therefore, lithium discontinuation studies may be enriched with patients responding to long-term treatment, in addition to tolerating treatment with lithium. While this type of study may tend to overestimate the efficacy of the enriched drug in Kaplan-Meier survival analyses with respect to all patients with bipolar disorders (Bowden et al. 2000; Gyulai et al. 2003), in meta-analyses, with categorical data used for analyses, the consequences may be harder to predict (see above). In addition, in the case of lithium, those randomized to placebo may be at an increased risk of a new affective episode (Suppes et al. 1991; Suppes et al. 1993) if lithium is rapidly discontinued following randomization (Severus et al. 2008; Coxhead et al. 1992). While we excluded lithium discontinuation studies as described above, we included studies enriched for tolerability to lithium (Prien et al. 1973) or acute response/tolerability to other agents (Calabrese et al. 2003; Bowden et al. 2003; Weisler et al. 2011). Therefore, we cannot exclude some form of bias in the comparison of lithium with lamotrigine, though the respective studies were primarily enriched for good tolerability to lamotrigine (Calabrese et al. 2003; Bowden et al. 2003). Furthermore, we do not know whether patients doing well on either quetiapine (Weisler et al. 2011) or lamotrigine (Calabrese et al. 2003; Bowden et al. 2003) will do better or less well on long-term lithium treatment compared to an unselected sample of remitted patients with bipolar disorders. Lithium proved superior to anticonvulsants in the prevention of manic episodes, while there was no significant difference regarding the prevention of depressive episodes, overall mood episodes, drop-out for reasons other than a mood episode, or study completers. However, the same limitations which applied to the interpretation of mutually exclusive events discussed above with regard to lithium versus placebo also apply. Nevertheless, the fact that lithium might do better than anticonvulsants regarding manic episodes while there was no significant difference regarding depressive episodes may be related to lamotrigine being the alternative treatment in three of the active comparator studies included in our analyses (Licht et al. 2010; Calabrese et al. 2003; Bowden et al. 2003). Lamotrigine has demonstrated its efficacy (Calabrese et al. 2003; Bowden et al. 2003) and has been granted approval in the European Union and the US for the long-term treatment of (bipolar I) depressive episodes and additional evidence being present for acute antidepressant properties (Geddes et al. 2009). However, there is no evidence supporting its use in the acute treatment of manic episodes and only limited evidence in the long-term treatment of manic episodes (Goodwin et al. 2004). Another issue which needs to be discussed is that in two of the included studies, the study population was exclusively enriched for patients being stable and tolerating lamotrigine for a period of several weeks (Calabrese et al. 2003; Bowden et al. 2003). While this methodological approach may confer some benefit for demonstrating efficacy in individual studies for the enriched agent using Kaplan-Meier survival analyses (Bowden et al. 2000; Gyulai et al. 2003), the consequences in a meta-analytical approach may be harder to predict as better tolerability may lead to fewer drop-outs due to reasons other than a mood episode and eventually more patients at risk to develop a mood episode. When we analyzed the data separately for lithium versus lamotrigine (Licht et al. 2010; Calabrese et al. 2003; Bowden et al. 2003), lithium was superior to lamotrigine in the prevention of (hypo)manic episodes, while lamotrigine did better than lithium in discontinuation for reasons other than a mood episode, with no significant difference between all other outcome parameter (data not shown). Taken together, and similar to the acute treatment of mania with anticonvulsants (Rosa et al. 2011), our data argue against the idea of a class effect of anticonvulsants in the prevention of depressive and manic episodes in bipolar disorders. Finally, in two of the included studies, the majority of patients had been on lithium in the past before entering the trial - in contrast to the active comparator (Calabrese et al. 2003; Bowden et al. 2003). As previous use of lithium during a patient’s lifetime has been found to be a risk factor for depressive episode (Severus et al. 2010), it would be desirable to only include lithium-naive patients in approval-seeking trials for a new compound, if lithium is used as active comparator.

It is unclear how far lithium benefits unselected patients with mood disorders in real-life clinical practice. Some studies have found poorer outcomes in clinical settings than would be anticipated from the results of the randomized evidence (Markar and Mander 1989). Some trials have attempted to replicate real-world conditions in their choice of inclusion/exclusion criteria, use of placebo, frequency of study visits, and lithium monitoring (Geddes et al. 2010). However, it remains unknown to what extent the results apply to the average clinical setting because the percentage of patients approached for initial evaluation of eligibility and those who participate in the trials is not routinely reported (Toerien et al. 2009; Schulz et al. 2010). Two important areas relating to the use of lithium in patients with bipolar disorders in clinical practice are not addressed here since they have been recently systematically reviewed elsewhere - prevention of suicide and physical health effects. Taken together, the available evidence shows that lithium is effective in the prevention of suicide and death from all causes in patients with mood disorders (Cipriani et al. 2013). Lithium use is associated with increased risk of reduced urinary concentrating ability, hypothyroidism, hyperparathyroidism, and weight gain; however, there is little evidence for a clinically significant reduction in renal function in most patients, and the risk of end-stage renal failure is low (McKnight et al. 2012). Nevertheless, long-term safety has not been adequately addressed in the studies upon which our meta-analysis is based as the studies included only covered a time span of up to 2 years. Finally, while our meta-analysis formally deals with the prevention of mood episodes, in studies where emergent mood episodes were the outcome measure, some of those may not have reached full syndromal criteria (Geddes et al. 2010). However, in clinical practice, the prevention of subsyndromal symptoms may be of comparable importance, though the literature available on this issue with regard to lithium is more limited (Frye et al. 2006).

#### Limitations at review level

The results of this meta-analysis have to be interpreted in the context of the methodology we used to conduct this study - and which we described in detail in the ‘Methods’ section. For example, we limited our meta-analysis to studies published either in English or German, although only one study was excluded for reasons of language (Figure 1). We employed the Cochrane Central Register of Controlled Trials (CENTRAL) and a thorough strategy to identify both published and unpublished studies, but it remains possible that estimates of effect may be affected by publication bias.

## Conclusions

In this meta-analysis, lithium is superior to placebo regarding prevention of overall mood episodes, manic episodes, completion of study (no mood episode and no drop out due to reasons other than a mood episode), and, dependent on the type of analyses performed, depressive episodes, while placebo is superior to lithium regarding drop out due to reasons other than a mood episode. With respect to the comparison with anticonvulsants, lithium is superior regarding prevention of manic episodes; however there is no significant difference regarding overall mood episodes, depressive episodes, drop-out due to reasons other than a mood episode, or study completion. With no other drug available having such ample and consistent evidence for its efficacy in the long-term treatment of bipolar disorders, lithium remains the most valuable treatment option in this indication (Miura et al. 2014).
